# Exposing sequence learning in a double-step task

**DOI:** 10.1007/s00221-016-4566-z

**Published:** 2016-02-12

**Authors:** Leonie Oostwoud Wijdenes, Eli Brenner, Jeroen B. J. Smeets

**Affiliations:** Research Institute MOVE, Department of Human Movement Sciences, VU University Amsterdam, 1081 BT Amsterdam, The Netherlands; Institute of Neurology, University College London, Box 146, Queen Square House, Queen Square, London, WC1N 3BG UK; Donders Institute for Brain, Cognition and Behaviour, Radboud University Nijmegen, Nijmegen, The Netherlands

**Keywords:** Motor learning, Sequence, Double-step, Movement corrections, Implicit learning

## Abstract

Is it possible to learn to perform a motor sequence without awareness of the sequence? In two experiments, we presented participants with the most elementary sequence: an alternation between two options. We used a double-step pointing task in which the final position of the target alternated between two quite similar values. The task forced participants to start moving before the final target was visible, allowing us to determine participants’ expectations about the final target position without explicitly asking them. We tracked participants’ expectations (and thus motor sequence learning) by measuring the direction of the initial part of the movement, before any response to the final step. We found that participants learnt to anticipate the average size of the final step, but that they did not learn the sequence. In a second experiment, we extended the duration of the learning period and increased the difference in size between the target position changes. Some participants started anticipating the step size in accordance with the sequence at some time during the experiment. These participants reported having noticed the simple sequence. The participants who had not noticed the sequence did not move in anticipation of the sequence. This suggests that participants who did not learn this very simple sequence explicitly also did not learn it implicitly.

## Introduction

With practice, people can learn complicated movement sequences. For example, dancers can remember long series of different postures and movements over time (Bläsing et al. 2012). Even when one is not explicitly trying to learn a sequence, performance changes after repeatedly performing the same series of movements. Sequence learning has been studied using a variety of tasks. One of the most popular ones is the serial reaction time task (Nissen and Bullemer 1987; Schwarb and Schumacher 2012). In this task, participants are typically instructed to place four fingers on four keys beneath four lamps and to press on a key as soon as the lamp above that key lights up (Nissen and Bullemer 1987). The order in which the lights appear could contain regularities. Using this task, Nissen and Bullemer (1987) found that the decrease in response time during a session was stronger for repetitions of the same sequence of key presses than for random series of presses, although participants had no declarative knowledge of the sequence. This has been interpreted as evidence for implicit motor learning of sequences (Nissen and Bullemer 1987; Willingham et al. 1989; Willingham and Goedert-Eschmann 1999; Robertson 2007). This interpretation was supported by findings that response times increased when a repeated sequence was replaced by a different sequence, or a random series of stimuli (Willingham et al. 1989; Shanks and Johnstone 1999; Wilkinson and Shanks 2004; Abrahamse et al. 2009).

However, it is not completely certain that the results of Nissen and Bullemer (1987) are the result of implicit motor learning of the sequence (Moisello et al. 2009; Shanks 2010; Krakauer and Mazzoni 2011; Dale et al. 2012). A possible complication is that declarative knowledge of a complex sequence after the experiment might not fully reflect what participants have learnt explicitly, because awareness of regularities in a complex sequence is probably a continuous process rather than a dichotomy, so reports could be influenced by the participants’ confidence or aptitude to mention impressions (Hannula et al. 2005; Robertson 2007). Moreover, the decrease in response time that is found in serial reaction time tasks could result from a combination of a reduction in movement time related to visuomotor learning of the task as such, and declarative knowledge of parts of the sequence (Moisello et al. 2009). Other measures of implicit learning than declarative knowledge of the sequence, such as reproducing (parts of) the motor sequence, or determining whether participants recognize parts of the sequence, all show a relation between learning and awareness (Shanks 2010).

Here, we sought to develop a behavioral measure with which we could monitor the state of sequence learning on a trial-by-trial basis without explicitly asking participants about the sequence. In the serial reaction time task, the finger is already at the response location (i.e., the key) at the onset of the trial. Other sequence learning paradigms introduce a distance between the initial position of the finger and that of the target (Moisello et al. 2009; Dale et al. 2012). This enables one to distinguish between the reaction time, the initial movement direction and the movement time. In these paradigms, decreases in the movement time could arise from task-related visuomotor learning, but the initial movement direction will only anticipate the upcoming target if participants have learnt the sequence. In these tasks, participants either move from a center position to different targets at equal distances from the center position in various directions (Moisello et al. 2009) or move between fixed targets (Dale et al. 2012). In both cases, any movements before (or just after) the next target appears reflect where the participant expects the next target to appear.

A problem with these paradigms is that if people do not initiate their movement before the target appears, it is impossible to determine where they expect it to appear. One can only presume that in such trials, participants had no expectations about where the target would appear, or at least that they were not sure enough about their expectations to rely on them to initiate movements. To evaluate the extent to which participants learn a sequence, it would be useful to be able to examine participants’ predictions about the upcoming target position on every trial. We therefore developed a paradigm with which we can monitor participants’ expectations about the next target on every trial, so that we can dissociate task-related visuomotor learning from sequence learning throughout the experiment. With this paradigm, we can monitor the development of trial-by-trial expectations even before participants learnt the sequence. On every trial, the initial movement direction reflects what participants implicitly learnt about the sequence.

We exposed participants to a sequence based on a double-step paradigm (Georgopoulos et al. 1981; Pélisson et al. 1986; Oostwoud Wijdenes et al. 2011). In this paradigm, the target that triggers the movement sometimes jumps to another position when participants start moving. Previous studies showed that if participants can predict the changes in target position or orientation (because the same change occurs on every trial), they adjust their initial movement direction in anticipation of the change (Fan et al. 2006; Bock et al. 2008; Schmitz et al. 2010). In our experiments, we studied participants’ behavior during blocks of trials in which the target appeared at the same position on every trial and always jumped to a new position when participants started their movement. The choices of new positions followed a simple sequence. Because the final target was only presented after the participants started to move, the initial movement direction reflects a prediction about the upcoming final target position. We examined whether participants would learn a simple sequence of final target positions without being provided with explicit information about any regularity in the order of the positions. We used the simplest sequence possible: an alternation between two conditions (ABAB…).

## Experiment 1

### Methods

#### Participants

Twelve participants (5 males and 7 females) aged 25–44 years (mean 30 years) who were naïve with respect to the research question agreed to participate voluntarily. They were right-handed and had normal or corrected-to-normal visual acuity. Informed consent was obtained from all participants included in the study. This study is part of a program that has been approved by the ethics committee of the faculty of Human Movement Sciences.

#### Experimental set-up

The experimental set-up was the same as the set-up used in Oostwoud Wijdenes et al. (2011). The set-up consisted of a 120 × 90 cm back-projection screen (Techplex 150, acrylic rear projection screen; tilted backward by 30°) and a projector that projected onto this screen (InFocus DepthQ Projector; resolution: 1024 × 768 pixels; screen refresh rate: 100 Hz). A marker was attached to the nail of the right index finger, and its position was registered with an Optotrak 3020 position sensor (500 Hz) that was located to the left of the screen. We use *horizontal* to refer to the left–right direction (positive to the right) and *vertical* to refer to the up-down direction along the screen (so positive is both up and away).

Optotrak recordings and stimulus presentation on the screen were controlled in MATLAB with the Optotrak Toolbox (Franz 2004) and the Psychophysics Toolbox (Brainard 1997). The Optotrak recordings and the stimulus presentation on the screen were synchronized using a photodiode. At the same time as the target presentation, we presented light to the photodiode that was connected to an Optotrak marker via a custom-built electronic circuit. The light resulted in deactivation of the Optotrak marker, so that marker deactivation marked the onset of the stimulus presentation on the screen. To hide the outline of the photodiode that was located at the upper left corner of the screen, the top 10 cm of the screen was covered.

#### Experimental design

**Fig. 1:**
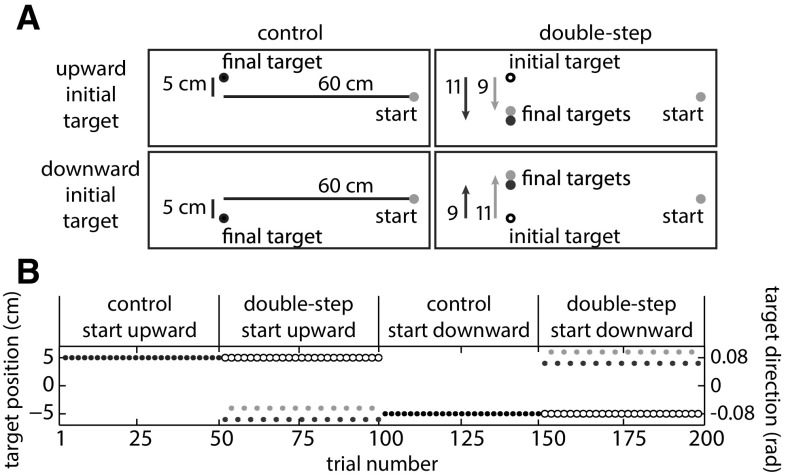
Design of experiment 1. **a** Illustration of the four different experimental blocks. **b** One of the possible time courses of the experiment, including the sequence of alternations between 9- and 11-cm target jumps

The experiment consisted of two blocks of 50 control trials and two blocks of 50 double-step sequence learning trials. The control and sequence learning blocks were presented in alternation, with the first block always being a control block. All trials had the same starting position: a pink dot with a radius of 1.5 cm, located 30 cm to the right of the screen center. In control trials, a 1.5-cm-radius, pink target dot appeared at the initial position and remained there. There were two initial target positions, both located 60 cm to the left of the starting position. One was also 5 cm downward along the screen and the other also 5 cm upward along the screen (Fig. 1a). The initial target position was the same throughout a block of control trials and the following block of sequence learning trials. In each block of double-step sequence learning trials, there were two possible final target positions. These positions were at the same horizontal position as the initial target, but they were either 9 or 11 cm higher or lower. They were always above the lower initial target position and below the higher initial target position. Whether the experiment started with the higher or lower initial target position was counterbalanced across participants. The size of the jump in the first double-step trial, 9 or 11 cm, was also counterbalanced across participants. Note that the average target position after the jump within each double-step block corresponds to the initial target position in one of the control blocks.

#### Procedure

Participants were standing in front of the screen and moved their finger from the starting position on the screen to the target position on the screen. During the movement, they were free to lift their finger off the screen. They were instructed to move to the target as quickly and as accurately as possible, but not to start moving before the target appeared. They were not informed about the possibility of target jumps or the presence of blocks with different trials. A random interval (0.5–1.5 s) after they put their finger on the starting position the target appeared at its initial position. In order to reduce the variation in reaction times, we presented a beep 23 ms before the target appeared. A finger displacement in the direction of the target of more than 1 mm triggered the target jump in the double-step blocks. To motivate participants to do their best, they received points for every hit target. The number of points they received was larger when the response time was faster. If they did not hit the target, they received no points. After the experiment, we asked participants whether they had noticed any order in the jumps.

#### Data analysis

In total, there were 2400 trials (12 participants; 200 trials each). Trials were excluded if (1) the marker was missing for more than 1 sample; (2) there was no registration of the moment of the target jump due to technical failure; (3) participants initiated their movement before the beep; (4) an error in the online finger displacement detection caused the target to jump before the real movement initiation (see next paragraph for definition of movement initiation); or (5) the screen was not hit. If there was one sample missing in a trial, we reconstructed its position with a cubic spline interpolation.

For the off-line analysis, the moment of movement initiation was defined as the last moment before the first peak in the tangential velocity at which the tangential velocity was lower than 0.02 m/s. The end of the movement was determined with the multiple sources of information (MSI) method (Schot et al. 2010). For this, the tangential velocity, horizontal position and elapsed time were converted into probability distributions. For the tangential velocity, the probability of a moment being the end of the movement scaled linearly from a value of 1 when the finger was not moving to a value of 0 at maximum tangential velocity. For the horizontal position, the probability of a moment being the end of the movement was 1 when the position was within 5 cm of the middle of the target and 0 when the position was outside this range. For the elapsed time, the probability of a moment being the end of the movement decreased linearly from a value of 1 at the moment of the target jump to a value of 0.9 at the end of the trial. This last distribution ensured that we took the first moment in time after the movement ended if participants stopped moving after hitting the target. The three probability distributions were multiplied, and the moment of the peak of the resulting distribution was considered to be the end of the movement.

The reaction time was the time between when the target initially appeared and movement initiation. The movement duration was the time between movement initiation and movement end. Motor learning has been shown to increase the symmetry of the velocity profile (Beggs and Howarth 1972; Nagasaki 1989). We therefore analyzed movement symmetry: the time to peak velocity expressed as a percentage of the total movement time.

We determined the initial direction in which the finger was moving (*aim direction*) as a measure of where the participant anticipated the final target to be. The initial movement direction is determined more reliably when the finger has covered more distance, so participants’ expectations are best revealed as late as possible, but obviously this must be determined before any online movement adjustments in response to the target jump could have occurred. Previous experiments in a similar set-up showed that participants can start to adjust their movement in response to the target jump 100 ms after the jump (Oostwoud Wijdenes et al. 2011). We therefore determined the direction in which the finger had moved 100 ms after the target jump (or after the moment the target would have jumped in the control blocks) to judge where the participant expected the target to be at the end of the trial. Our measure of direction (*aim direction)* was the angle that the finger’s displacement made with the horizontal when projected onto the plane of the screen, where 0 corresponds to a displacement to the left, and a positive angle to a deviation upward (Fig. 1b). A similar measure at the end of the movement (considering the whole displacement) indicates the angle at that time. We will refer to this as “final direction.”

In order to be able to average the *aim* and *final direction* across participants, despite the counterbalanced orders of initial target positions and jump sizes, the trial numbers were interchanged to align the configurations. For participants who started the experiment with the lower initial target position, trials 1–100 were changed into trial numbers 101–200, and vice versa. For participants who started the first double-step block with the smaller jump size, the numbers of consecutive even and odd trials were swapped within the double-step blocks. We will refer to the trial numbers after all these changes as *adjusted trial numbers*. The trial numbers were not adjusted before averaging the reaction time and movement time, because we expected these measures to depend more on the experimental progression than on the exact location of the target. In order to test whether performing the experiment affected a certain movement parameter over time, we determined the average behavior for different phases. *Early* behavior was the average of the first 20 trials of a block, and *late* was the average of the last 20 trials of a block.

To examine whether participants timed their movements differently when targets jumped, we compared the average reaction times and movement times in the control blocks and the double-step blocks with paired-samples *t* tests. To examine whether performing the sequence influenced the reaction time and movement time within the double-step blocks, we performed 2 × 2 repeated-measures ANOVAs with within participant factors block number (first double-step block or second double-step block) and phase (early or late). In addition, we examined whether there were changes in movement symmetry over the course of the experiment with a 2 × 2 × 2 repeated-measures ANOVA with within participant factors block number (first or second block of each type), block type (control or double step) and phase (early or late).

We used two paired-samples *t* tests to examine whether participants learnt to adjust their initial movement direction to the average target position after the jump. We compared the average *aim direction* early in the first control block, with the *aim direction* late in the last double-step block. We did the same for the second control block and the first double-step block. Most importantly, to examine whether participants learnt to anticipate the sequence, we averaged the *aim direction* for the lower and higher targets late in the double-step blocks and determined whether the *aim direction* for the lower and higher final targets was different with a paired-samples *t* test. Additionally, we tested whether the subjects used information of the previous trial and thus whether *aim direction* on trial *i* was related to the *final direction* on trial *i* − 1. To do this, we performed linear regression analyses for the early and late phases of the double-step blocks within subjects and blocks, averaged over the blocks. A one-sample *t* test tested whether the early slopes or late slopes were different from zero. A positive slope would indicate that the aim was biased toward the previous final direction (de Lussanet et al. 2001); a negative slope would indicate that the aim was biased toward the upcoming target position, and thus would show anticipation of the sequence.

### Results

**Fig. 2:**
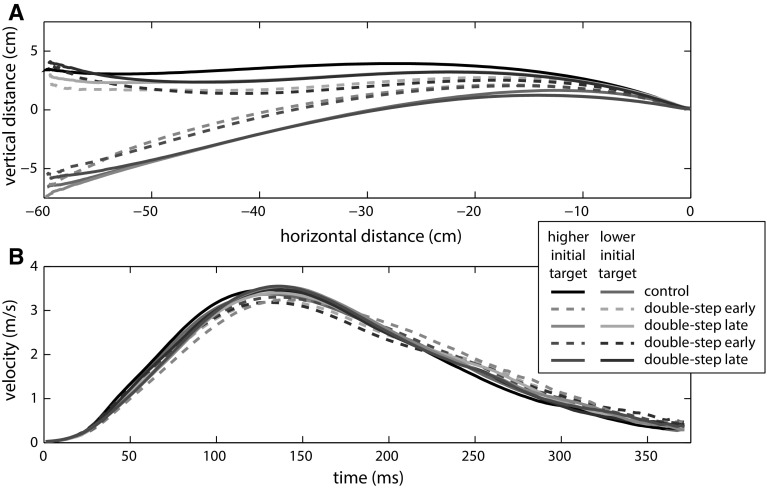
Average movement trajectories for experiment 1. **a** Movement paths as projected on the screen. **b** Tangential velocity. Data for both control and double-step blocks. Trials early (*dashed lines*) and late (*solid lines*) during the double-step blocks are averaged separately

A total of 86 of the 2400 trials were excluded from further analysis, 2–24 trials (1–12 %) per participant. On average, the target jumped 53 ms after what we defined off-line to have been the moment of movement initiation. This is as close as we could get due to inevitable delays in the set-up (~20 ms) and limitations in the online detection of movement initiation. None of the participants reported having realized that there was an order in the target jumps after the experiment. Figure 2 shows the average position and velocity profiles.

**Fig. 3:**
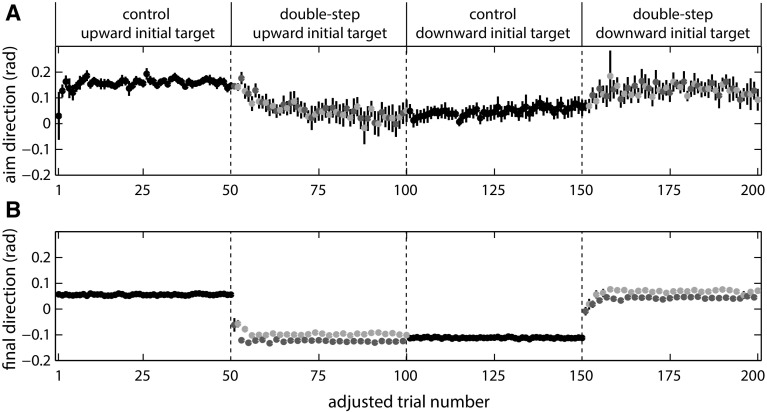
Spatial aspects of the results of experiment 1. Aim direction (**a**) and final direction (**b**) for each adjusted trial number, averaged across participants. Aim direction was determined 100 ms after the target jump. *Error bars* indicate ±1 SE. *Color coding* of the trials as in the previous figures (control trials in *black*; double-step higher final target positions in red; double-step lower final target positions in *blue*). The adjustment to the trial number means that the order in the figure does not match the trial order in the experiment: Half of the participants actually performed the trials with the downward initial target before the trials with the upward initial target (for details, see “Methods”) (color figure online)

Figure 3a shows the *aim direction* for each trial, averaged across participants. In the late phase, there was no significant difference in *aim direction* between the control blocks and the sequence learning blocks that had the same average final target position (blocks 1 and 4 *p* = 0.374; blocks 2 and 3 *p* = 0.434). Thus, participants adjusted their *aim direction* in anticipation of the target jumps. Notice that on average, the *aimdirection* is mostly larger than 0 (i.e., upward), which is probably related to the observation that movement paths were slightly curved, with participants on average approaching the target from above (see Fig. 2a).

We saw that participants learnt to adjust their initial movement direction in anticipation of the jumps. But did they anticipate the sequence, or only learn the average jump size? For the late phase of the double-step blocks, the *aim direction* did not differ significantly between targets that jumped to lower and higher positions [*t*(11) = 1.00, *p* = 0.338; mean value of 0.08 rad both for the lower targets and for the higher targets]. Notice that with respect to the starting position, the lower final target directions are −0.1 and 0.07 rad, and the higher final target directions are at −0.07 and 0.1 rad. If participants had learnt the sequence, we would have expected them to have a lower average *aim direction* for targets that jumped to lower positions than for targets that jumped to higher positions.

Figure 3b shows the direction in which the finger had moved by the end of the movement, averaged across participants. This *final direction* shows that participants did not completely correct for the target jump in the first trials of the double-step blocks, but matched the size of their correction to the size of the jump after a few trials. The relation between the *aim direction* on trial i and the *final direction* on trial *i* − 1 was not significantly different from zero, neither early [mean 0.17; *t*(11) = 0.81, *p* = 0.435] nor late during the double-step blocks [mean −0.06; *t*(11) = 0.51, *p* = 0.617]. Thus, our participants did not learn to anticipate the sequence.

**Fig. 4:**
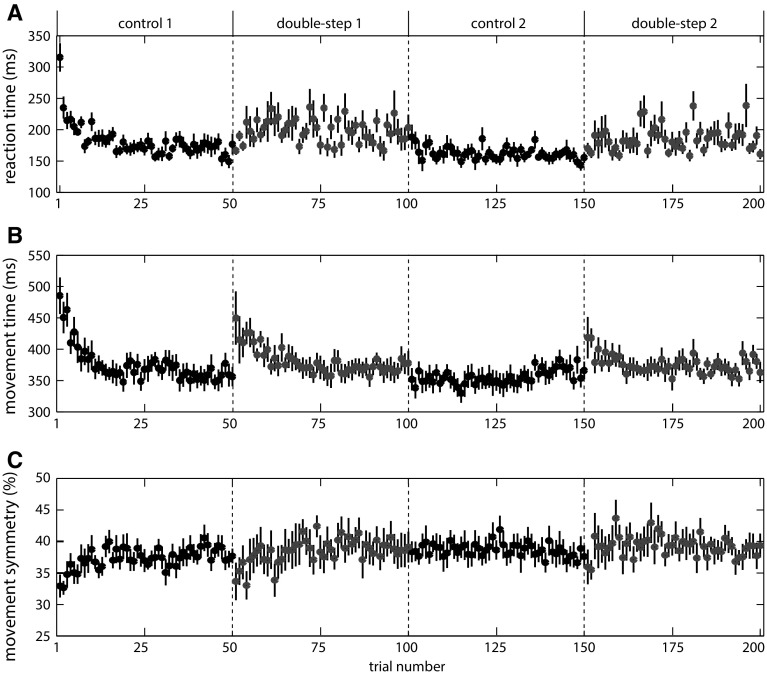
Temporal aspects of the results of experiment 1. Reaction time (**a**), movement time (**b**) and movement symmetry (**c**) during the course of the experiment, averaged across participants, irrespective of target position for control (*black*) and double-step trials (*purple*) (color figure online)

Figure 4a shows the reaction time, averaged across participants. Center-out task studies interpreted a decrease in reaction time as sequence learning (Ghilardi et al. 2003; Moisello et al. 2009). In our set-up, the two information sources that triggered the initial response, the beep and the target appearing at its initial position, were independent of the sequence. So, even if participants had learnt the sequence, we would not expect such learning to decrease reaction times. Paired-samples *t* tests revealed that the reaction time in control blocks (mean 172 ms) was significantly shorter than the reaction time in double-step blocks [mean 192 ms; *t*(11) = 4.58, *p* = 0.001]. However, the 2 × 2 repeated-measures ANOVA did not reveal main effects for block number or phase, or an interaction effect, so repeating the sequence did not influence the reaction time over the course of the experiment or within the double-step blocks.

In the absence of a change in *aim direction*, we interpret a decrease in movement time and an increase in movement symmetry as evidence for learning the visuomotor task (Fig. 4b, c). The movement time (mean 371 ms) was not significantly different between control and double-step trials. The 2 × 2 repeated-measures ANOVA revealed a significant main effect for phase [*F*(1,11) = 10.64, *p* = 0.008]. The movement time was shorter for late (mean 370 ms) than for early trials (mean 389 ms) within the double-step blocks. There was no main effect for block number, and no interaction effect.

The 2 × 2 × 2 repeated-measures ANOVA on the movement symmetry showed a significant main effect of block number [*F*(1,11) = 9.85, *p* = 0.009]. Movements in the second control/double-step block (mean 39 %) were slightly more symmetric than movements in the first block (mean 38 %). The interaction between block number and phase was also significant [*F*(1,11) = 7.85, *p* = 0.017], because the symmetry increased within the first occurrence of a block (means 37 % for early; 39 % for late trials; averaged across both kinds of trials), but did not change within the second occurrence (both means 39 %). There were no other main or interaction effects, suggesting that the changes in movement symmetry were not different between control and double-step trials.

### Discussion

Reaction times were longer in the double-step blocks than in the control blocks (Fig. 4a). Within double-step blocks, there was no change in reaction time with experience. Serial reaction time studies found that the response time, a combination of reaction time and movement time, decreased over the experiment if the targets were presented in a systematic order (Nissen and Bullemer 1987; Shanks and Johnstone 1999). Earlier attempts to separately investigate reaction time and movement time interpreted a decrease in reaction time as evidence for sequence learning (Ghilardi et al. 2003; Moisello et al. 2009). In those studies, participants were allowed to start moving before the target appeared, because such early movements were critically necessary to show participants’ anticipation of the sequence. In our experimental paradigm, participants were not allowed to start moving before the target appeared, and the final target only appeared after movement initiation. Our reaction time is therefore not informative of sequence learning and not comparable to reaction times in previous studies. Possible reasons for the reaction time being longer in double-step blocks might be that participants tended to delay their reaction in the hope that the target would have jumped before they started moving, or that they increased their threshold for movement initiation because they had to correct their movement in the previous trial, or that estimating the anticipated final target position took some additional time.

We did not find a difference in movement time between control and double-step blocks, but the movement time did decrease within several blocks and the movement symmetry increased during the first control and double-step blocks (Fig. 4b, c). In center-out sequence learning tasks, movement duration increased once participants became aware of the sequence (Ghilardi et al. 2003; Moisello et al. 2009). Moisello et al. (2009) argue that the increase in movement duration (together with a decrease in response time) reflects an energy-saving strategy to optimize movement time and endpoint accuracy. They interpreted a gradual decrease in movement duration before participants became aware of the sequence as evidence for learning the visuomotor task. Our participants did not report having noticed any order in the target jumps at the end of the experiment. There was also no indication that they had learnt the sequence in their *aim direction*. Probably, the observed decrease in movement time and the increase in movement symmetry within blocks reflect visuomotor learning for the average 60 cm movement, rather than for the specific small (2 cm) differences in the vertical component.

Over the course of the double-step blocks, the *aim direction* gradually shifted toward the average position of the target after the jump (Fig. 3a). This is consistent with previous studies in showing initial movement direction adjustments to targets that jumped on every trial (Bock et al. 2008; Schmitz et al. 2010). The shift that we see in Fig. 3a seems to stabilize by the time about half of the trials in a block were performed. This justifies our choice of using the last 20 trials of each block to analyze sequence learning.

We did not find evidence of sequence learning for a simple two-element sequence in our double-step paradigm. The participants’ movement endpoints did depend on the sequence, but this was achieved by correcting the movements online to end at the final target position (Fig. 3b).

Since we were specifically interested in *implicit* learning of a motor sequence, we intentionally exposed participants to each sequence for a limited time and kept the difference between the two final target positions quite small. Since none of the participants detected the sequence *explicitly*, it should be possible to give participants more chance to learn the sequence *implicitly* by increasing the number of repetitions of the same sequence and increasing the difference between the sizes of the second steps. We therefore performed a second experiment in which the difference in size between the two jumps was substantially larger and the duration of the learning blocks was extended.

## Experiment 2

### Methods

Twelve other naïve participants (4 males and 8 females) aged 23–34 years (mean 27 years) participated voluntarily in experiment 2. Again, they were all right-handed and had normal or corrected-to-normal visual acuity, and gave their informed consent.

**Fig. 5:**
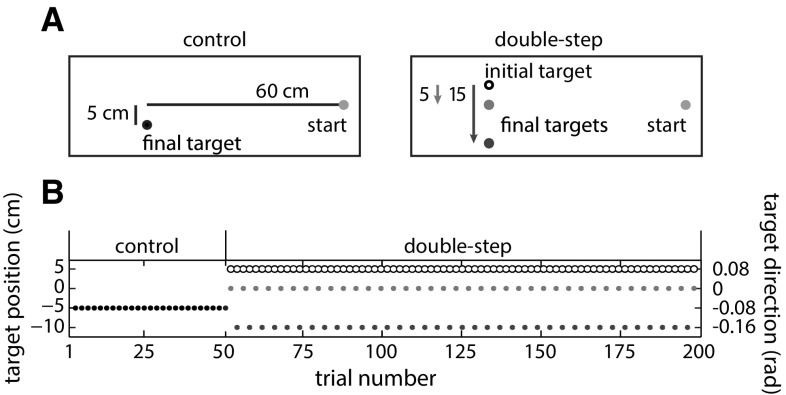
Design of experiment 2. (**a**) Illustration of the two different experimental blocks. (**b**) One of the two possible time courses of the experiment, starting the sequence of alternations with a 5-cm target jump

The experimental set-up, the procedure and the data analysis of the second experiment were exactly the same as for the first experiment, only the experimental design was different. There were now only two blocks: one control block of 50 trials and one double-step block of 150 trials. The target in the control block was always 5 cm lower than the starting position along the screen (Fig. 5). In the double-step block, the initial target position was always 5 cm higher than the starting position along the screen. The two final target positions were 5 cm lower than the initial target position (at the same height as the starting position) and 15 cm lower than the initial target position (so that the average final position in the double-step block was the same as in the control block). The experiment started with 50 control trials, followed by 150 double-step sequence learning trials in which the target alternated between jumping 5 cm downward and 15 cm downward. The size of the first target jump was counterbalanced across participants. In order to average data across participants despite the counterbalanced order of jump sizes, trial numbers were interchanged to align the configurations. For participants who started with the 15-cm jump, numbers of consecutive odd and even trials were swapped.

### Results

A total of 88 out of 2400 trials were excluded from further analysis, 2–23 trials (1–11.5 %) per participant. Despite the substantial difference in jump sizes (10 cm), only five of the twelve participants (2 males) reported having noticed the sequence when asked after the experiment. Because the participants who noticed the sequence performed clearly differently from the participants who did not notice the sequence, we do not report the results of statistical tests across all participants.

**Fig. 6:**
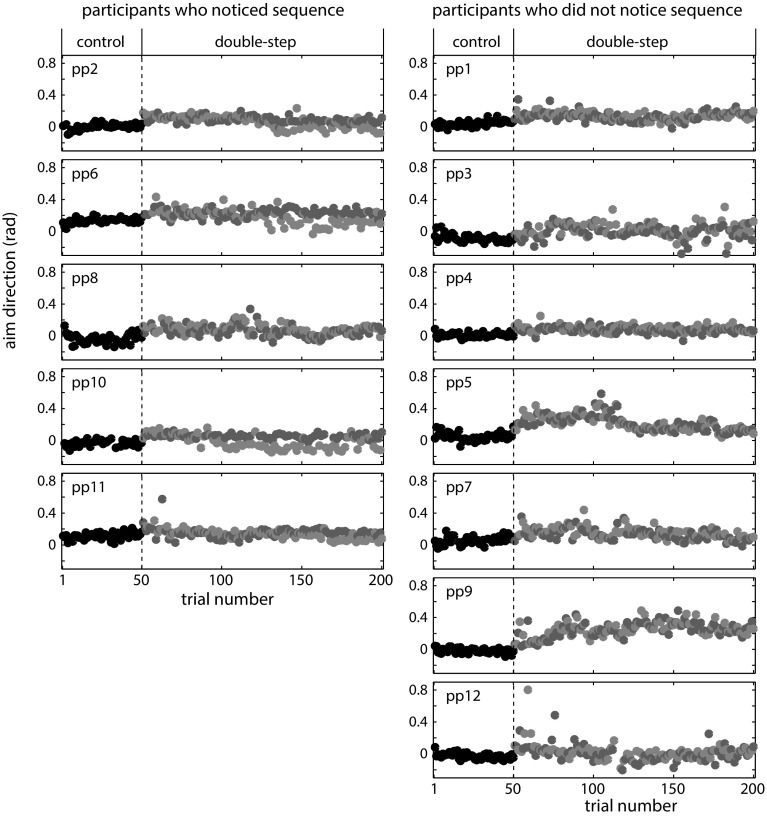
Aim direction for individual participants who noticed the sequence (*left*) and who did not notice the sequence (*right*). *Color coding* as in Fig. 5 (color figure online)

**Fig. 7:**
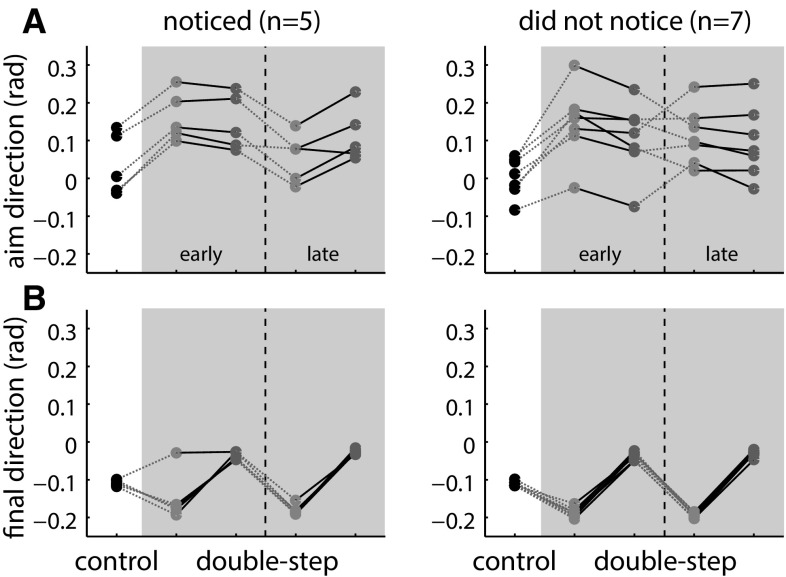
Spatial aspects of the results of experiment 2. Aim direction (**a**) and final direction (**b**) for control trials (*black*), lower final target positions trials (*blue*) and higher final target position trials (*red*), for participants who noticed (*left*) and did not notice the sequence (*right*). The lines connect data points of the same participant (color figure online)

Figure 6 shows the *aim direction* for each trial, both for participants who did notice the sequence (left) and for those who did not notice the sequence (right). The average *aim* and *final direction* are shown in Fig. 7. For the participants who noticed the sequence, a clear difference between the *aim direction* for the two jump sizes emerged after between 90 and 160 trials (Fig. 6, left column, blue and red dots diverge). An exception is participant 8, who reported only having noticed the sequence on the last few trials. No difference between the aim for the two jump sizes emerged for the participants who did not notice the sequence. The earliest indication of sequence learning can be observed in participant 10. He started to show differences in *aim direction* about 40 trials after the beginning of the double-step block, thus after 20 repetitions of the sequence. For both groups, the average *aim direction* in the double-step block seems to be higher than the average *aim direction* in the control trials. Thus, on average participants underestimated the size of the jump.

**Fig. 8:**
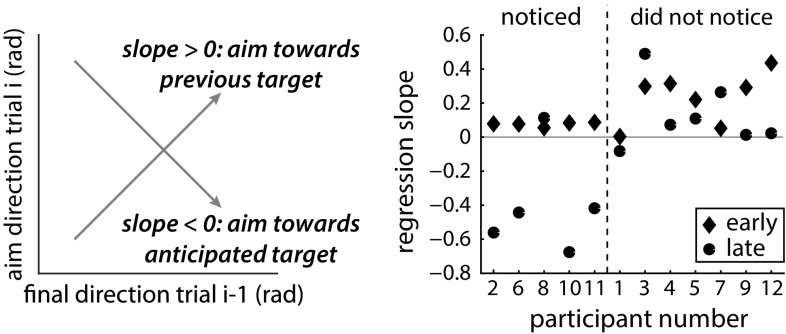
Relationship between the aim direction on the current trial and the final direction on the previous trial early and late during the double-step block

For participants who noticed the sequence, the relation between the *aim direction* on the current trial and the *final direction* on the previous trial was somewhat positive at the start of the double-step block and became negative by the end of the block (except for participant 8, see Fig. 8). In contrast, most of the participants who did not notice the sequence maintained to show positive values for this relationship throughout the double-step block. Some of the participants who did not notice initially had larger positive values than the participants who noticed, indicating that the participants who did not notice had a larger tendency to adjust their *aim direction* to match the position on the previous trial. This means that they were not just generally slower to learn. One might think that the participants who did not notice the sequence relied more on the previous target position to determine where to aim their current movement, and had to unlearn this tendency first (shift of the slope toward zero) before they could start learning to anticipate the sequence. Indeed, 5 of the 7 participants had a lower value for the regression slope at the end of the double-step block. These values were near zero, as one would expect if participants neither adjust their *aim direction* to the position on the preceding trial nor learnt the sequence, so they had unlearnt the tendency. The abrupt changes in the *aim direction* that we see in Fig. 6 for the participants who did notice the sequence suggest that the change to a negative value was primarily a consequence of noticing the sequence rather than of gradually unlearning the tendency to rely on the previous trial.

**Fig. 9:**
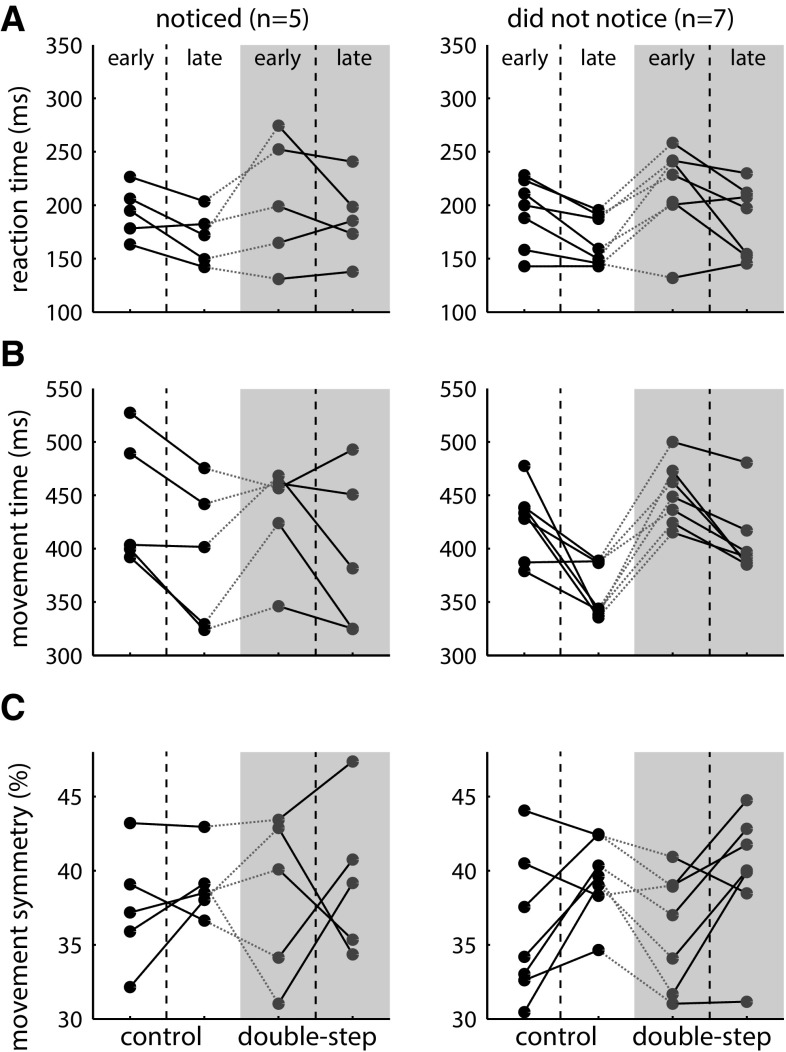
Temporal aspects of the results of experiment 2. Reaction time (**a**), movement time (**b**) and movement symmetry (**c**) for control trials (*black*) and double-step trials (*purple*), for participants who noticed (*left*) and did not notice the sequence (*right*). Separate data points show the measures for the early and late phases of the control and double-step blocks (color figure online)

Reaction time and movement time became shorter during the first control block for both groups of participants (Fig. 9a, b black dots). Reaction time and movement time increased at the start of the double-step block and then decreased again. Most participants show an increase in movement symmetry within the blocks, although in both groups there are some participants who show a different pattern (Fig. 9c). The general decrease in movement time and increase in movement symmetry within the blocks suggest that participants learnt the visuomotor task.

### Discussion

Despite a very clear difference in the size of the alternating jumps, only five of the twelve participants noticed this simple sequence within the 150 double-step trials. Four of these five participants showed clear anticipation of the sequence (Fig. 6, left column). The fifth (participant 8) reported only having noticed that there was a sequence during the last trials. All the participants who did not notice the sequence show a similar *aim direction* for both jumps (Fig. 6, right column). We can therefore conclude that these participants did not, implicitly or explicitly, learn the sequence in this experimental paradigm. Only participants who had explicit awareness of the sequence showed signs of sequence learning.

In a study using center-out movements, with movement time as the measure of visuomotor learning of the task, movement times increased when the sequence was learnt (Moisello et al. 2009). This was interpreted to be the result of an energy-saving strategy. We did not observe an increase in movement time when participants anticipated the sequence (Fig. 9b). However, in our paradigm, a long movement duration might save energy as long as the sequence has not been learnt. If participants do not anticipate the target jump correctly, they have to adjust their movement online. Moving more slowly means that the finger will have moved less far by the time the adjustment takes place, so that presumably the required adjustment will be smaller and therefore cost less energy. Moreover, although responding to target jumps of up to 5 cm early during the movement need not increase the overall movement duration (Turrell et al. 1998; Liu and Todorov 2007; Oostwoud Wijdenes et al. 2011), we might expect movement durations to increase after 15 cm movement adjustments. If so, learning the sequence might decrease movement time, because the movement can go directly to the target.

## General discussion

Serial reaction time studies suggest that people can learn complicated sequences without explicit awareness of the sequence. However, in the double-step paradigm that we examined, only a few participants learnt the simple sequence, and those who learnt the sequence also noticed that there was a sequence. Thus, we find no evidence of implicit sequence learning.

All participants that showed a clear difference in *aim direction* for the two sequence elements were aware of the sequence. This is consistent with earlier reports of a relation between sequence learning and awareness (Shanks and Johnstone 1999; Wilkinson and Shanks 2004; Moisello et al. 2009; Shanks 2010; Moisello et al. 2011). These findings support the view that knowledge is important for motor learning (Stanley and Krakauer 2013; Wong et al. 2015).

Our sequence was so simple that we expected participants to start anticipating the upcoming final target position after a few repetitions, as we would expect for a key press sequence that alternates between two keys (although that sequence would probably immediately be noticed). However, most of our participants did not even anticipate the sequence after 75 repetitions. Moreover, it took participants who learnt the sequence at least 20 repetitions to start anticipating, while Nissen and Bullemer (1987) found sequence learning for a much more complicated 10-trial sequence after only 6 repetitions of the sequence [although other studies suggest—in line with our results—that 25–40 repetitions of the sequence are needed (Willingham et al. 1989; Shanks and Johnstone 1999; Wilkinson and Shanks 2004)]. Moreover, the participants who learnt the sequence all reported being aware of it, so it is quite likely that their performance followed their awareness of the sequence.

We were mainly interested in evidence for implicit learning, so it is quite fortunate for our purpose that participants often did not explicitly learn the sequence. We do not know why it was so difficult for participants to learn this. Most likely, it has nothing to do with the sequence itself, as much more complicated sequences can be learnt without specific instructions (Nissen and Bullemer 1987; Willingham et al. 1989; Shanks and Johnstone 1999; Willingham and Goedert-Eschmann 1999). It might have to do with the small difference between the two final target positions in experiment 1, but this cannot explain the lack of sequence learning for more than half of the participants in experiment 2. Maybe the lack of sequence learning for the majority of participants is related to the fact that the sequence was in a perturbation. In response to force field perturbations (Shadmehr and Mussa-Ivaldi 1994), 168 trials was not enough to learn to anticipate a sequence of two alternating opposing force fields while moving between three targets in the absence of contextual cues about which field was present (Karniel and Mussa-Ivaldi 2003).

Another possibility is that time is an important factor for implicit sequence learning. In serial reaction time studies, a new element of the sequence is typically presented every 500 ms. The intervals for other learning paradigms, such as the center-out task and the continuous-tracking task, are often somewhat longer, with a new element presented about every second (Chambaron et al. 2006; Moisello et al. 2009, 2011; Lang et al. 2013). In our paradigm, a new initial target was presented approximately every 8 s. A fast succession of elements might be important for sequence learning. Also, the time interval between successive sequence elements was not fixed, which has been shown to hinder learning (Lang et al. 2013).

Although most of our participants did not learn the sequence, all of them learnt to anticipate the average size of the target jump. This is consistent with earlier findings showing that movement planning is influenced by movements made in previous trials (de Lussanet et al. 2001; Scheidt et al. 2001; de Lussanet et al. 2002; van Beers 2009) and that people do anticipate target changes that occur consistently on every trial (Fan et al. 2006; Bock et al. 2008; Schmitz et al. 2010). Besides anticipation of the average target jump, decreases in reaction time and movement time and increases in movement symmetry support the conclusion that participants learnt to optimize their motor performance. The lack of motor learning during the experiment seems to be specific for the sequence. In conclusion, online movement corrections do influence the way the next movements are planned. However, movement plans only consider the sequence if participants noticed that there was a sequence. We did not find evidence for implicit sequence learning with the double-step paradigm.
